# Effects on Quality of Application of Two Antagonistic Yeasts on Plums (*Prunus salicina*) During Postharvest Cold Storage

**DOI:** 10.3390/foods14173101

**Published:** 2025-09-04

**Authors:** Paula Tejero, Alicia Rodríguez, Alberto Martín, Carlos Moraga, Emilio Aranda, Alejandro Hernández

**Affiliations:** 1 Nutrición y Bromatología, Escuela de Ingenierías Agrarias, Universidad de Extremadura, 06007 Badajoz, Spain; 2 Instituto Universitario de Investigación en Recursos Agrarios (INURA), Universidad de Extremadura, Avd. de la Investigación, 06006 Badajoz, Spain

**Keywords:** biocontrol, shelf-life, spoilage, colour, postharvest

## Abstract

Plums are climacteric fruits with a short postharvest shelf-life, which makes them highly susceptible to spoilage by moulds and pathogens. Biological control using antagonistic yeasts offers a promising approach to extend shelf-life by inhibiting fungal growth. This study evaluated the effects of two yeast strains, *Hanseniaspora uvarum* L793 and *Metschnikowia pulcherrima* L672, on the quality of ‘Larry Ann’ Japanese plums during cold storage. Plums were divided into three batches: two treated by immersion in yeast suspensions (10^8^ cells mL^−1^) and one untreated control. Quality parameters assessed over 12 weeks at 1 °C included weight loss, decay index, microbial counts, yeast colonisation, skin and flesh colour, texture, pH, titratable acidity, total soluble solids, and ripening index, with evaluations every week. *M. pulcherrima* L672 showed strong colonisation and persistence on the plum surface, significantly reducing skin damage and mould incidence. In contrast, *H. uvarum* L793 initially colonised well but declined over time, being replaced by native yeasts such as *Aureobasidium* spp. Both treatments maintained the physicochemical and organoleptic quality of the plums throughout storage. However, *M. pulcherrima* L672 was more effective in suppressing fungal growth and preserving fruit integrity. These findings suggest that *M. pulcherrima* L672 is a promising biocontrol agent for prolonging the shelf-life of Japanese plums during cold storage, maintaining their commercial quality for up to three months.

## 1. Introduction

Plums are one of the most widely consumed stone fruits in the world. From a commercial perspective, the most widely traded plums belong to European plum (*Prunus domestica* L.) and Japanese plum *(Prunus salicina* Lindl.) species, generally marketed under different names [1]. The FAO (Food and Agriculture Organization of the United Nations) [2] reports that the Asian continent produced 66% of plums in 2023, with Europe following with 21.9%. As for the most important countries in plum production, China was the leading producer with more than 6.9 million tonnes, followed by Romania and Serbia with 645,090 and 422,935 tonnes, respectively.

Consumers value plums for their health benefits related to antioxidant and anti-inflammatory activities and proven effects on memory improvement [3]. The varieties and crop management affect their nutritional composition [4]. However, plums typically contain main sugars like sucrose, glucose, sorbitol, and fructose. Malic acid is the most notable acid found in this fruit. Plums are also rich in functional polyphenols, including phenolic acids and flavonoids [5,6].

Nevertheless, the postharvest shelf-life of plums is limited. The main factors that limit the shelf-life of plums during marketing are over-ripening and fungal decay [7]. Pathogenic and spoilage fungi that affect plums during ripening and commercialisation and cause significant economic losses include *Mucor* sp. [8], *Monilinia* sp. [8,9,10], *Botrytis cinerea* [8,10], and *Penicillium* sp. [8,11], among others. Fruit loss due to the presence of mould is a significant problem, especially in developing countries, where losses can be between 20% and 50%. In developed countries, the loss is lower, ranging from 5% to 35% [12,13,14]. This difference mainly comes from variations in postharvest handling and available technology. Improving storage and handling practices, together with the use of non-chemical solutions, could be key to reducing global food waste [12,13,14]. Plums have undergone different treatments to help delay ripening, limit fungal development, and extend shelf-life. Chemical treatments with 1-MCPD [15] and nitric oxide [16] can modulate ethylene production, which delays ripening. Furthermore, edible coatings are an effective strategy for reducing transpiration in plums and extending their shelf-life. Several studies show that various edible coatings can enhance the post-harvest quality of plums. Mixtures of pectin and carboxymethylcellulose applied to cv. ‘Golden Drop’ improved parameters such as firmness, bioactive compound content, and antioxidant capacity [17]. Waxy maize starch high in amylopectin (Versasheen ^®^) is used in the ‘Jojo’ and ‘Tophit Plus’ varieties. It reduces mass loss and keeps firmness and extends post-harvest shelf-life [18]. Similarly, rice starch-ι-carrageenan reduced weight loss and respiration rate. This helps keep the fruit quality high [19]. The alginate coating on the ‘Blackamber’, ‘Larry Ann’, ‘Golden Globe’, and ‘Songold’ cultivars inhibited ethylene production. This delayed ripening and extended the optimal storage time by two to three weeks compared to the controls [20]. Likewise, similar effects were obtained in plums (*P. salicina* L.) cv. ‘Black Diamond’ by combining chemicals (40 mg L^−1^ of ClO_2_ for 10 min) and physical treatments (100 W of ultrasound for 10 min) to improve storability [21]. The use of putrescine is another effective method for reducing ethylene production [22]. In addition, essential oils, such as thymol, can control the development of *Monilia fructicola* in plum trees cv. ‘Violette’ and ‘Veeblue’ [23].

Biological control offers a promising, non-chemical strategy for extending fruit shelf-life by suppressing fungal growth. The literature describes numerous applications of antagonistic microorganisms on different stone fruits, with a focus on the action mode of yeasts. Effective antifungal treatments for peaches, nectarines, and plums include *Debaryomyces hansenii* and *Wyckerhamomyces anomalus* [24,25], *Pichia membranaefaciens* and *Kloeckera apiculata* [26,27], *Aureobasidium pullulans* [28,29], and *Candida pseudolambica* [30]. These help to counteract the growth of *Monilia* sp., *B. cinerea*, and *P. expansum*. These investigations determine the antagonism mechanisms of action and the efficiency of antagonistic yeasts against pathogenic moulds using confrontation trials on damaged fruit. However, the influence of yeast treatments on fruit quality has received little attention. The primary goal of this research was to determine the effect of two antagonistic yeasts (*Metschnikowia pulcherrima* L672 and *Hanseniaspora uvarum* L793) on the quality of Japanese plums of the cv. ‘Larry Ann’.

## 2. Materials and Methods

### 2.1. Biological Material

In this study, Japanese plums (*Prunus salicina)* of the cultivar ‘Larry Ann’ were used after being stored at 1 °C (ideal refrigeration temperature) for up to 77 days. At first, they displayed values of 17.4 ± 0.8 °Brix, 2.53 ± 0.11 g malic acid/100 g fresh weight, a pH of 3.55 ± 0.10, and a texture of 11.38 ± 1.25 N/mm.

Plums were hydrocooled with water containing 100 μg L^−1^ of sodium hypochlorite at 1–4 °C for 3 min. The fruits were then rinsed in sterile water for 2 min and completely air-dried at room temperature. The fruits were then treated with two antagonistic yeasts: *H. uvarum* L793 [31,32], whose mechanism of antagonism is based on the production of antifungal volatile organic compounds (VOCs), and *M. pulcherrima* L672 [33,34,35], whose mechanism of action is primarily based on competition for nutrients. Prior to this, the yeasts were cultivated on agar potato dextrose (PDA) medium for 48 h at 25 °C. Next, using an orbital shaker, a loop was collected for inoculation in yeast extract peptone dextrose broth (YPD broth) at 25 °C/48 h and 100 r.p.m. After centrifugation at 5400× *g* for 5 min, the yeast cells were washed twice with phosphate-buffered saline (PBS) and then resuspended in 100 mL of the same solution. Cell concentration was determined by microscopic observation in a Neubauer chamber.

### 2.2. Yeast Inoculation and Cold Storage of Plums

Three batches of yeasts were prepared for the assay: a control batch with no yeast inoculation (control), a batch with the yeast *M. pulcherrima* L672 (L672), and a batch inoculated with the yeast *H. uvarum* L793 (L793). The plums were inoculated by immersing the yeasts in a solution of cold water (5 °C) with a yeast concentration of 10^8^ cells mL^−1^. In the control batch, only cold water was used. After treatment, the plums were dried at room temperature. Then, plums were packed in half-kilo plastic punnets of fruit, which were sealed with a macroperforated film with 6 holes of 9 mm diameter. Finally, they were stored in a cold room at 1 °C and 95% relative humidity in the dark. Three punnets were taken weekly for analysis. The assay was repeated twice.

### 2.3. Lab Analysis

#### 2.3.1. Weight Loss

Every punnet from every batch was weighed after it was packed. The punnets chosen at random for sampling from each batch were weighed once a week. Lastly, the following formula was used to determine the weight loss:

(1) $$\%\;\mathrm{weight}\;\mathrm{loss}=100-\left[\left(\frac{W_{i}}{W_{0}}\right)\times 100\right]$$

where W_i_ represents the weight of each punnet at the different sampling times, and W_0_ represents the weight of the punnet after packaging.

#### 2.3.2. Microbiological Analysis: Determination of Decay Incidence, Microbial Analysis, and Inocula Monitoring

In each sampling, 12 fruits per sample (36 fruits per batch) were observed and inspected for external appearance. The fruits were observed for mould growth, rot, brown or abnormal spots, softening or any other characteristics that indicate change or deterioration. With these data, the decay incidence was determined as the percentage of fruit with symptoms of microbiological spoilage [33].

By using microbiological seeding on acidified PDA medium, the number of mould and yeast was determined. For each sample, three fruits were analysed. Using a sterile scalpel, a 4 cm^2^ square of skin was cut off each fruit. Following homogenisation, the skin samples were homogenised in 10 mL of peptone water, and decimal dilutions were prepared using the same culture medium. After seeding 100 mL of the medium in acidified PDA medium, the plates were incubated at 25 °C for 5 days. Yeast and mould colonies were counted independently, and the results were expressed as log_10_ CFU/cm^2^.

Yeast monitoring was performed by isolating 5 colonies from each sample with the characteristic morphologies of the inoculated species. Once isolated, DNA extraction was performed using the Quick-DNA Fungal/Bacterial Miniprep KIT (Zymo Research). These DNAs were used to amplify the 26S large subunit of the rDNA following the method described by Ruiz-Moyano et al. [36]. Finally, all PCR products were purified using the Genejet PCR Purification Kit (Thermo-Fisher Scientific, Waltham, MA, USA) and the PCR products were sequenced in both directions at the Service of Bioscience Applied Techniques (STAB) of the University of Extremadura (Badajoz, Spain). The two resultant sense and antisense sequences were edited with Chromas Pro version 1.49 beta (Technelysium, South Brisbane, Australia), and a consensus sequence was built and compared with the European Molecular Biology Laboratory (EMBL) nucleotide sequence and GenBank databases, using the BLAST algorithm + 2.16.0. The identities of the isolates were determined based on the highest score (>99%).

#### 2.3.3. Physicochemical Analysis

##### Colour Measurement

A colour analysis was carried out on both the skin and the flesh of the plums. This analysis was carried out using a Konica Minolta CM-600d digital colorimeter connected to a computer with the Spectramagic program. The colour was determined using the CIELAB colour space, giving the a* (from red to green), b* (from blue to yellow), and L* (luminosity) values. Chromaticity (C*) and hue (H*) were calculated from the above values using the following formulae:

(2) $$h^{*}=\tan^{-1}\left(\frac{b^{*}}{a^{*}}\right)$$

(3) $$C^{*}=\sqrt{{\left(a^{*}\right)}^{2}+{\left(b^{*}\right)}^{2}}$$

Five fruits were measured for each sample (15 fruits per batch and sampling day).

##### Firmness

Texture was determined using a TA.XT2i texture analyser (Stable Micro Systems, Godalming, UK). The instrument was fitted with a 30 kg load cell (Stable Micro Systems, Godalming, UK) and a probe SMS P/50. A compression test was performed, with a pre-test speed of 5 mm/s, a test speed of 0.20 mm/s, and a post-test speed of 10 mm/s. The applied strain was 6%.

Five fruits were measured for each sample in duplicate, for a total of 10 measurements per batch.

##### Total Soluble Solids, pH, and Titratable Acidity

Total soluble solids (TSS), pH, and titratable acidity (TA) were measured for each independent homogenate (*n* = 3) per batch. Three fresh pitted fruits were used for each measure and homogenised using an Omni Mixer homogeniser (Omni International, Marietta, GA, USA).

The TSS values were measured using an automatic temperature-compensated DR101 digital refractometer (Optic Ivymen System, Barcelona, Spain). Results are expressed as °Brix.

TA and pH were determined for each homogenate in 2 g aliquots diluted to 18 mL with de-ionised water from a Milli-Q water purification system (Millipore, Bedford, MA, USA). Analyses were conducted using a 716 DMS Titrino automatic titrator (Metrohm, Herisau, Switzerland). Samples were titrated with 0.1 M NaOH up to pH 8.1. Results are expressed as g malic acid per 100 g fresh weight (FW).

The ripening index was calculated from the ratio of TSS/TA [37,38].

### 2.4. Data Analysis

Statistical analysis of the data of the present study was performed using the SPSS statistical package for Windows (IBM Corp. SPSS Statistics, Version 22, Armonk, NY, USA). The data corresponding to % weight loss, microorganism count (moulds/yeasts), % damage, colour, pH, titratable acidity, °Brix, and firmness were analysed. An analysis of variance (ANOVA) was conducted to observe the effect of the different treatments over the days. When there were significant differences in the means, a Tukey test was carried out (*p* ≤ 0.05). In addition, PCA was performed to relate physicochemical and microbiological parameters involved in plums’ quality in the three plum batches (control, batch inoculated with *M. pulcherrima* L672, and batch inoculated with *H. uvarum* L793) during 28 and 77 days of cold storage.

## 3. Results and Discussion

### 3.1. Microbiological Analysis Results

**Figure 1:**
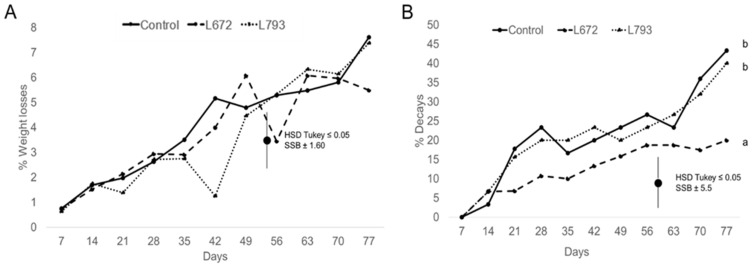
(**A**) Weight loss (%) of *Prunus salicina* L. cv. ‘Larry Ann’ treated with *Metschnikowia pulcherrima* L672, *Hanseniaspora uvarum* L793, and the control batch during a 77-day storage period at 1 °C. (**B**) Percentage of decay incidence of the *Prunus salicina* L. cv. ‘Larry Ann’ treated with *M. pulcherrima* L672, *H. uvarum* L793 and the control batch during a 77-day storage period at 1 °C. Different letters indicate significant differences (*p* ≤ 0.05) among batches over the entire period.

Antagonist yeast treatments had no significant (*p* > 0.05) effect on plum weight loss. The data showed an evolution in weight loss from values below 1% after one week of cold storage to values around 7% after 77 days of cold storage (Figure 1A). These results coincide with those reported by Zhao et al. [39], who observed that the application of *Debaryomyces hansenii* did not produce significant differences in the rate of weight loss, although their study was conducted at 25 °C and during a 4-day storage period. In contrast, Íñiguez-Moreno et al. [40] demonstrated that the antagonistic yeast *Meyerozyma caribbica* significantly reduced weight loss in avocados stored under refrigeration for 15 days. This suggests that this parameter can be maintained or even improved depending on the biocontrol yeast used and the type of fruit to which it is applied.

The first symptoms of decay were detected on day 14 of cold storage, with values in all treatments of around 6% (Figure 1B). A decrease in decay incidence was noted in plums treated with *M. pulcherrima* L672 starting on day 21 (*p* < 0.05). These plums showed a progressive increase in the percentage of decayed fruit to a mean value of 22.30%. The application of the yeast *H. uvarum* L793 did not reduce the decay incidence, with similar values to the control group. At the end of the assay (day 77 of cold storage), both batches presented mean percentages of decayed fruit of 50.00% and 46.36%. These results are consistent with those described by Xu et al. [41] who investigated the effect of 1-methylcyclopropene on the quality of *Prunus salicina* cv. ‘Taoxingli’ plums. Decay incidence began to be observed on day 18 in the control group, with values below 10%, and increased periodically throughout the storage period.

**Figure 2:**
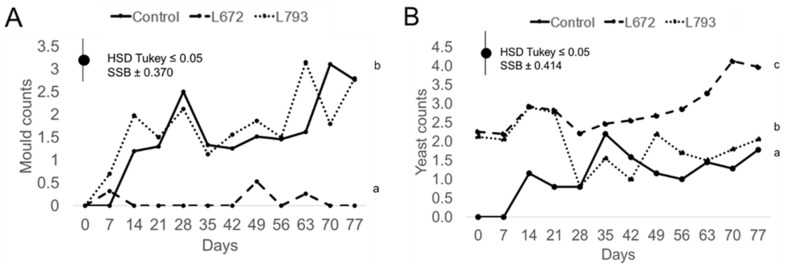
Counts of moulds (**A**) and yeast (**B**) in Log_10_ CFU/cm^2^ of *Prunus salicina* L. cv. ‘Larry Ann’ treated with *Metschnikowia pulcherrima* L672, *Hanseniaspora uvarum* L793, and the control batch during a 77-day storage period at 1 °C. Different letters indicate significant differences (*p* ≤ 0.05) among batches over the entire period.

Plums treated with *M. pulcherrima* L672 differed from the other two batches in terms of mould counts (Figure 2A). During the experiment, mean mould counts were less than 1 log_10_ CFU/cm^2^ when the L672 yeast was used. After 77 days of cold storage, the counts of the control batch and the batch treated with *H. uvarum* L793 rose to 2.76 ± 0.58 and 2.80 ± 0.00 log_10_ CFU/cm^2^, respectively. Similarly, after the eighth week of storage at 1 °C, Rocha-Pimienta et al. [42] found that ‘Larry Ann’ plums had higher mould counts. On the other hand, regarding *H. uvarum* L793, even though no significant differences were seen in the decrease in moulds, Ruiz-Moyano et al. [31] found that this yeast reduced the incidence of *B. cinerea* in strawberries, while Tejero et al. [32] found that the strain had an antifungal impact on *Aspergillus flavus*. Since the primary antifungal mechanism of this yeast involves the production of antifungal volatile organic compounds (VOCs) [31,32], its lack of efficacy may be attributed to its low capacity to colonise the plum surface, thus not generating enough VOCs to manifest this antagonistic effect.

Regarding yeast counts, the batches that were inoculated with yeasts showed comparable counts of yeast of up until day 21 of cold storage (Figure 2B). In the batches treated with *M. pulcherrima* L672 and *H. uvarum* L793, yeast counts were initially 2.3 ± 0.00 log_10_ CFU/cm^2^ and 2.1 ± 0.00 log_10_ CFU/cm^2^, respectively; in the control batch, yeast counts were below the detection limit due to the initial disinfection of the plum surface. After 21 days, the batches inoculated with *M. pulcherrima* L672 and *H. uvarum* L793 had higher yeast counts (2.8 ± 0.12 log_10_ CFU/cm^2^ and 2.82 ± 0.12 log_10_ CFU/cm^2^, respectively), whereas the control batch had lower counts (0.79 log_10_ CFU/cm^2^). On day 28, the *H. uvarum* L793-treated batch’s yeast counts dropped to 0.80 ± 0.00 log_10_ CFU/cm^2^, which was comparable to the control batch. Yeast counts in the control and L793-treated batches were comparable starting on day 28. After 77 days of cold storage, the control batch and that treated with *H. uvarum* L793 had counts of around 2 log_10_ CFU/cm^2^, while the batch inoculated with *M. pulcherrima* L672 yeast had an increase in yeast counts to 3.98 ± 0.61 log_10_ CFU/cm^2^. The yeast *M. pulcherrima* L672 had the highest yeast counts in the study by Cabañas et al. [33], which used a combination of modified atmosphere packaging and various antagonistic yeasts on cherries (*Prunus avium* L.). On day 40 of storage at 2 °C, the yeast reached values ranging from 5 to 6 log_10_ CFU/g.

The morphologies of the yeast colonies in the plum samples inoculated with *M. pulcherrima* L672 exhibited the typical characteristics of this species, being surrounded by a red halo [43,44]. Colony identification by sequencing of the major 26S rDNA subunit confirmed the identity of these isolates in all identified colonies during storage. On the other hand, colonies with a morphology consistent with the *H. uvarum* species were no longer detected from day 28 of sampling onwards, even though most colonies isolated from plums inoculated with *H. uvarum* L793 had morphologies consistent with this species up until day 21. The molecular identification of the isolates confirmed the presence of *H. uvarum* for up to 21 days of cold storage. In subsequent samplings in this batch, only isolates of the genus *Aureobasidium* were found. The yeast population in the control batch was identified as *Aureobasidium* sp. based on the observed morphologies; no morphologies resembling those of *M. pulcherrima* or *H. uvarum* were found.

### 3.2. Physicochemical Analysis Results

**Figure 3:**
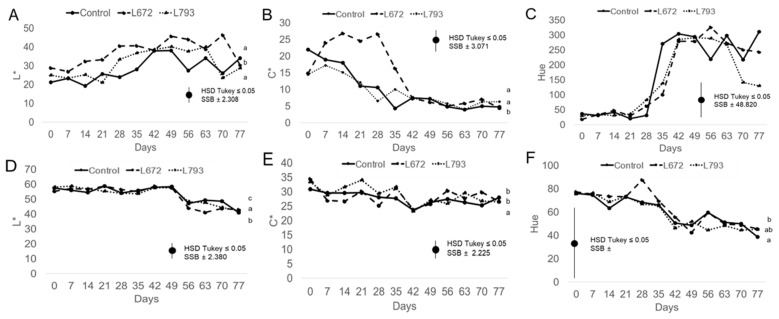
Plum skin colour values measured in L* (**A**), C* (**B**) and Hue angle (**C**) and flesh L* (**D**), C* (**E**) and Hue angle (**F**) of *Prunus salicina* L. cv. ‘Larry Ann’ treated with *Metschnikowia pulcherrima* L672, *Hanseniaspora uvarum* L793, and the control batch during a 77-day storage period at 1 °C. Different letters indicate significant differences (*p* ≤ 0.05) among batches over the entire period.

Figure 3 illustrates the effect of the treatments on plum skin (A, B, and C) and flesh colour (D, E, and F). Skin luminosity increased in all treatments during storage until sampling day 70 (Figure 3A). However, until sampling day 35, the *M. pulcherrima* L672 treatment resulted in higher luminosity values (*p* < 0.05) than the other two batches. The plums treated with *M. pulcherrima* L672 differed from the other plums in terms of skin chromaticity (C*; Figure 3B). The chromaticity values of plums from the L672 batch increased to 26.63 ± 3.50 units, while those of the control and *H. uvarum* L793-treated plums gradually decreased over the first 35 days of storage. Chromaticity levels were comparable among all batches after day 35. Ultimately, skin chromaticity values of approximately 5 units were displayed by all batches (Figure 3B). Bautista-Ortín et al. [45] studied the effect of two strains of commercial yeasts in winemaking, observing that they produced an increase in the colour intensity of the wines. In particular, the potential of *M. pulcherrima* strains to improve colour in wines has been observed in association with the formation of stable pigments [46,47]. Similarly, antagonistic strains of *M. pulcherrima* have been shown to delay skin colour changes in mango [48]. Finally, in terms of skin hue (Figure 3C), all treatments displayed a similar trend, with values increasing from day 35 of cold storage and remaining at approximately 250 units until the end of the assay. These results differ from those described by Diaz-Mula et al. [49], who observed that the hue value decreased progressively until day 35 at 2 °C in plums of the ‘Larry Ann’ variety. Martínez-Esplá et al. [50] also described a decrease in hue value over the post-harvest period in the ‘Black Splendor’ and ‘Royal Rosa’ varieties.

Regarding the parameters of plum flesh colour, there were no significant differences between treatments (*p* > 0.05; Figure 3D–F). During storage, flesh luminosity (Figure 3D) decreased slightly, from approximately 56 to approximately 42 units by the end of the assay. Flesh chromaticity (Figure 3E) displayed relatively stable values during storage, from mean values of 32 units at the start to values of 28 units at 77 days. Finally, the flesh hue (Figure 3F) colour decreased from 77 to 42 units on average. A decrease in all of the plum colour parameters, both in the flesh and the skin, is a typical trend over time [20,51]. However, it can occasionally be observed that some parameters, like the skin’s hue and L*, increase over time because cold storage can enhance these values [52].

Regarding firmness (Table 1), fruit firmness decreased across all treatments, ranging from 11.38 ± 2.11 to 15.23 ± 5.14 N/mm on day 0, to values between 5.59 ± 1.91 and 7.91 ± 2.84 N/mm on day 77. Consistent with these findings, Avci et al. [52] and Fawole et al. [53] reported that the firmness of *Prunus salicina* L. and African Delight™ plums decreased during storage, up to week 5, at 0 °C and −0.5 °C, respectively. Although some significant differences were found at intermediate sampling points, the overall analysis indicated that firmness was not significantly influenced (*p* > 0.05) by the application of the antagonistic yeasts.

Regarding titratable acidity, significant differences (*p* ≤ 0.05) were found between the batch treated with *H. uvarum* L793 and the control batch, with the inoculated batch generally exhibiting higher values (Table 1). Throughout the storage period, the values of this parameter decreased from a range of 2.08 ± 0.10 to 2.53 ± 0.00 g malic acid/100 g FW to between 1.26 ± 0.06 and 1.42 ± 0.06 g malic acid/100 g FW on the final day. Similarly, a decline in this parameter during storage days was also reported in studies on plums conducted by Liu et al. [51] and Zhang et al. [54].

Regarding total soluble solids (Table 1), the batch treated with *H. uvarum* L793 exhibited lower mean values than the control batch and the batch treated with *M. pulcherrima* L672, indicating overall significant differences (*p* ≤ 0.05). Throughout the storage period, the trend of the yeast-treated batches remained relatively consistent. From day 0 to day 77, the values for the control decreased from 17.40 ± 0.14, which is the normal value for ‘Larry Ann’ plums, despite being high when compared to other varieties [55], to 15.87 ± 0.15 °Brix.

The ripening index (Table 2) revealed significant differences (*p* ≤ 0.05) in the batch treated with L793, which showed lower ripening values compared to the control, suggesting a potential delay in plum ripening. These values ranged from 8.27 ± 0.25 on day 0 to 12.25 ± 0.24 on day 77 of storage, whereas on the final day, the control values were 12.55 ± 0.56. The ripening index values increased from 8.28 ± 0.36 to 13.43 ± 0.60 in the batch treated with *M. pulcherrima* L672, but no significant differences (*p* > 0.05) were found between this batch and the control. According to Lozano et al. [55], the plums had low ripening index values at the start of storage, indicating they were not fully ripe. This is also related to texture, since the results of Lozano et al. [55] for ‘Larry Ann’ showed that the plum softens as the ripening index rises. These findings are also consistent with those reported by Valero et al. [38] and Xu et al. [41].

Throughout the storage period, pH values showed a slight upward trend overall (Table 2). Values increased from an initial range of 3.17 ± 0.08 to 3.56 ± 0.15 to a final range of 3.87 ± 0.03 to 3.95 ± 0.01. The batch treated with *M. pulcherrima* L672 showed significant differences compared to the control (*p* ≤ 0.05). However, the batch treated with *H. uvarum* L793 did not show significant differences (*p* > 0.050) compared to the control. These values fall within the range expected for plums [55,56].

These findings regarding physicochemical quality are consistent with other studies reporting that the application of *H. uvarum* and *M. pucherrima* on various fruits did not alter these parameters, thus preserving postharvest fruit quality [48,57,58].

### 3.3. Overall Impact on Plums Quality

**Figure 4:**
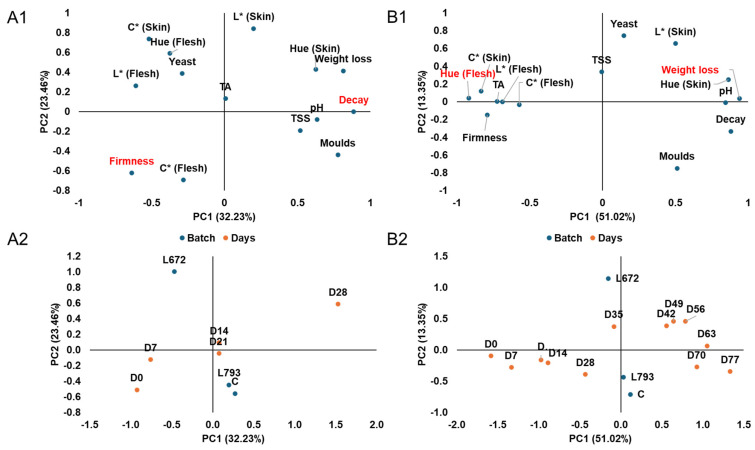
Loading plot (**A1**,**B1**) and score plot (**A2**,**B2**) after principal component analysis of physico-chemical and microbiological analysis of the three plums batches (control, batch inoculated with *Metschnikowia pulcherrima* L672 and batch inoculated with *Hanseniaspora uvarum* L793) during 28 and 77 days of cold storage, respectively, defined by the two first principal components (PC1 and PC2). Subfigures (**A1**,**B1**) highlight in red the variables with the highest significance in PC1, both on the positive and negative axes.

Figure 4 (A1,A2,B1,B2) illustrates the relationships among the physicochemical and microbiological characteristics of the plums, the treatment, and storage time over the first 28 (Figure 4(A1,B1)) and 77 days of storage (Figure 4(A2,B2)), respectively. The inoculation of *M. pulcherrima* L672 was positively correlated with yeast counts and the maintenance of colour parameters during the initial days of storage (D0 and D7), as shown in Figure 4(A1,A2), which was defined by principal components 1 (PC1) and 2 (PC2) and explained 55.69% of the total variability. Conversely, this yeast controlled the decay incidence and the presence of moulds. Its primary mechanism of action is competition for iron, which results in the synthesis of a compound known as pulcherrimin, which binds to free ions in the medium and limits their bioavailability. This effect is associated with its biocontrol capacity [43,59]. On the other hand, it is evident that the *H. uvarum* L793-yeast treatment was grouped with the control plums, indicating that yeast had a limited effect on the plum quality at 28 days.

The analysis of PC1 and PC2 (Figure 4(B1,B2)) demonstrates the relationship between physicochemical and microbiological parameters with treatments and storage days across the 77-day experiment, accounting for 64.37% of the total variability. The plum ripening process is evident, as seen by the positive correlation between the positive axis of PC1 and parameters such as darker skin tones, increased decay incidence, increased pH, and decreased moisture content with the final days of storage (D63, D70, and D77).

During the last days of storage, there was also a negative correlation with plum firmness, titratable acidity, and other colour characteristics. According to Singh et al. [16] and Li et al. [60], the physicochemical parameters evolved according to the natural evolution linked to the ripening and senescence processes in this particular fruit variety. Additionally, plums treated with *M. pulcherrima* L672 showed a positive correlation with yeast counts on the positive axis of PC2 at the end of storage, while mould counts and decay incidence showed a negative correlation.

Future research should examine the impact of these antagonistic yeasts on various plum varieties, since some studies, such as those by Díaz-Mula et al. [49] and Díaz-Mula et al. [61], found variations in the evolution of some physicochemical characteristics during storage in plums of different varieties, such as those with yellow and purple skin. On the other hand, it should be noted that a storage temperature of 1 °C was used to carry out this research, which is a temperature that is suboptimal for yeast growth. Even so, *M. pulcherrima* L672 was able to colonise the surface of the plums, so at higher temperatures, it will probably colonise more easily [62]. This will be tested in future studies by comparing the results obtained with different storage temperatures.

## 4. Conclusions

Controlling spoilage moulds in the postharvest stages of different fruits can be challenging for the industry, particularly in the context of minimising the use of synthetic chemicals and managing products in a sustainable manner throughout the marketing chain. Antagonistic yeasts represent a viable alternative to synthetic fungicides, contributing to a more sustainable food supply chain. Verification of their in vivo ability to control fungal decay development without compromising the quality of the products to which they are applied is a prerequisite for their successful implementation. This study evaluated the effect of two antagonistic yeasts, *M. pulcherrima* L672, whose mechanism of action is mainly associated with competition for nutrients, and *H. uvarum* L793, a potential producer of VOCs with antifungal activity, on ‘Larry Ann’ plums during 77 days of refrigerated storage. The findings indicate that *M. pulcherrima* L672 demonstrated a strong ability to control mould growth, significantly reducing decay incidence, but the yeast *H. uvarum* showed limited efficacy under the conditions evaluated in this matrix. Furthermore, the plums’ primary quality indicators were unaffected, positioning *M. pulcherrima* L672 as a promising candidate for development as a biofungicide.

## Figures and Tables

**Table 1:** Evolution of firmness, titratable acidity, and total soluble solids of the plums *Prunus salicina* L. cv. ‘Larry Ann’ treated with *Metschnikowia pulcherrima* L672, *Hanseniaspora uvarum* L793, and the control batch during a 77-day storage period at 1 °C.

	Cold Storage												
	D0	D7	D14	D21	D28	D35	D42	D49	D56	D63	D70	D77	Mean
	**Firmness (N/mm)**												
**Control**	11.38 ^b^ ± 2.11	15.13 ^a^ ± 5.18	11.21 ^a^ ± 1.79	9.98 ^a^ ± 2.12	10.18 ^a^ ± 3.34	7.19 ^a,b^ ± 3.28	6.49 ^a^ ± 1.87	7.05 ^b^ ± 1.17	5.01 ^b^ ± 1.22	8.14 ^a^ ± 2.09	7.70 ^a^ ± 1.88	5.59 ^a^ ± 1.91	8.75 ^a^ ± 3.71
**L672**	15.23 ^a^ ± 5.14	12.58 ^a^ ± 3.36	8.86 ^a^ ± 2.88	9.59 ^a^ ± 2.09	6.34 ^b^ ± 1.78	6.44 ^b^ ± 2.51	5.84 ^a^ ± 2.54	9.34 ^a^ ± 1.79	8.48 ^a^ ± 1.59	8.42 ^a^ ± 1.99	6.79 ^a^ ± 1.32	7.91 ^a^ ± 2.84	8.82 ^a^ ± 3.66
**L793**	14.20 ^a,b^ ± 1.76	14.96 ^a^ ± 4.19	10.25 ^a^ ± 4.05	10.46 ^a^ ± 2.89	9.30 ^a,b^ ± 3.94	9.90 ^a^ ± 2.56	6.09 ^a^ ± 1.31	5.19 ^c^ ± 0.95	6.82 ^a^ ± 1.92	7.80 ^a^ ± 1.55	6.85 ^a^ ± 3.10	6.11 ^a^ ± 3.58	8.99 ^a^ ± 4.07
*p*	*0.044*	*0.350*	*0.242*	*0.725*	*0.029*	*0.026*	*0.764*	*0.000*	*0.000*	*0.762*	*0.598*	*0.182*	*0.882*
	**TA (g Malic Acid per 100 g Fresh Weight (FW))**												
**Control**	2.54 ^a^ ± 0.00	1.68 ^a^ ± 0.38	1.84 ^a^ ± 0.07	1.90 ^a^ ± 0.12	2.03 ^a^ ± 0.06	1.63 ^b^ ± 0.08	1.66 ^b^ ± 0.07	1.96 ^b^ ± 0.02	1.45 ^b^ ± 0.03	1.32 ^a^ ± 0.09	1.43 ^a^ ± 0.05	1.27 ^b^ ± 0.05	1.70 ^a^ ± 0.34
**L672**	2.09 ^b^ ± 0.10	1.85 ^a^ ± 0.15	1.91 ^a^ ± 0.09	2.09 ^a^ ± 0.03	2.09 ^a^ ± 0.01	1.75 ^b^ ± 0.06	2.13 ^a^ ± 0.08	1.73 ^c^ ± 0.04	1.57 ^a^ ± 0.04	1.33 ^a^ ± 0.03	1.33 ^a,b^ ± 0.06	1.33 ^a,b^ ± 0.05	1.76 ^a^ ± 0.31
**L793**	2.09 ^b^ ± 0.06	1.98 ^a^ ± 0.09	1.78 ^a^ ± 0.02	1.94 ^a^ ± 0.17	2.13 ^a^ ± 0.13	2.18 ^a^ ± 0.14	1.99 ^a^ ± 0.12	2.09 ^a^ ± 0.08	1.59 ^a^ ± 0.03	1.38 ^a^ ± 0.04	1.22 ^b^ ± 0.04	1.42 ^a^ ± 0.03	1.80 ^a^ ± 0.33
*p*	*0.011*	*0.486*	*0.160*	*0.224*	*0.444*	*0.001*	*0.002*	*0.000*	*0.003*	*0.522*	*0.007*	*0.013*	*0.835*
	**TSS (°Brix)**												
**Control**	17.40 ^a^ ± 0.14	17.63 ^a^ ± 0.12	16.80 ^b^ ± 0.10	17.53 ^a^ ± 0.29	17.37 ^b^ ± 0.06	17.63 ^a^ ± 0.06	17.47 ^a^ ± 0.15	17.47 ^a^ ± 0.06	18.77 ^a^ ± 0.06	17.10 ^b^ ± 0.20	17.20 ^a^ ± 0.35	15.87 ^c^ ± 0.15	17.35 ^a^ ± 0.67
**L672**	17.25 ^a^ ± 0.07	16.73 ^b^ ± 0.25	16.50 ^c^ ± 0.10	16.87 ^b^ ± 0.06	17.80 ^a^ ± 0.00	17.83 ^a^ ± 0.12	17.53 ^a^ ± 0.06	17.30 ^a^ ± 0.44	17.37 ^c^ ± 0.23	17.80 ^a^ ± 0.17	17.73 ^a^ ± 0.06	17.83 ^a^ ± 0.21	17.38 ^a^ ± 0.48
**L793**	17.30 ^a^ ± 0.00	17.00 ^a,b^ ± 0.40	17.17 ^a^ ± 0.06	17.97 ^a^ ± 0.06	17.87 ^a^ ± 0.06	16.77 ^b^ ± 0.06	16.67 ^b^ ± 0.21	16.97 ^a^ ± 0.06	17.87 ^b^ ± 0.06	16.93 ^b^ ± 0.06	15.90 ^b^ ± 0.44	17.43 ^b^ ± 0.06	17.14 ^a^ ± 0.60
*p*	*0.372*	*0.020*	*0.000*	*0.001*	*0.000*	*0.000*	*0.001*	*0.127*	*0.000*	*0.001*	*0.001*	*0.000*	*0.201*

Different letters represent significant differences batches at the same cold storage time (*p* ≤ 0.05).

**Table 2:** Evolution of ripening index and pH of the plums *Prunus salicina* L.cv. ‘Larry Ann’ treated with *Metschnikowia pulcherrima* L672, *Hanseniaspora uvarum* L793, and the control batch during a 77-day storage period at 1 °C.

	Cold Storage												
	D0	D7	D14	D21	D28	D35	D42	D49	D56	D63	D70	D77	Mean
	**Ripening Index**												
**Control**	6.86 ^b^ ± 0.07	10.86 ^a^ ± 2.24	9.12 ^a,b^ ± 0.30	9.24 ^a^ ± 0.46	8.54 ^a^ ± 0.26	10.81 ^a^ ± 0.55	10.56 ^a^ ± 0.43	8.92 ^b^ ± 0.11	12.94 ^a^ ± 0.25	12.96 ^a^ ± 1.05	12.00 ^b^ ± 0.31	12.55 ^a^ ± 0.56	10.55 ^a^ ± 1.93
**L672**	8.28 ^a^ ± 0.36	9.07 ^a^ ± 0.64	8.67 ^b^ ± 0.40	8.08 ^a^ ± 0.13	8.53 ^a^ ± 0.03	10.22 ^a^ ± 0.42	8.23 ^b^ ± 0.29	9.98 ^a^ ± 0.19	11.06 ^b^ ± 0.39	13.39 ^a^ ± 0.20	13.39 ^a^ ± 0.62	13.43 ^a^ ± 0.60	10.25 ^a^ ± 2.10
**L793**	8.27 ^a^ ± 0.25	8.87 ^a^ ± 0.36	9.63 ^a^ ± 0.09	9.33 ^a^ ± 0.89	8.42 ^a^ ± 0.55	7.70 ^b^ ± 0.50	8.39 ^b^ ± 0.58	8.13 ^c^ ± 0.32	11.22 ^b^ ± 0.22	12.30 ^a^ ± 0.31	13.00 ^a,b^ ± 0.28	12.26 ^a^ ± 0.24	9.84 ^a^ ± 1.87
*p*	*0.0180*	*0.225*	*0.021*	*0.070*	*0.891*	*0.001*	*0.001*	*0.000*	*0.000*	*0.195*	*0.019*	*0.063*	*0.318*
	**pH**												
**Control**	3.56 ^a^ ± 0.15	3.57 ^a^ ± 0.05	3.44 ^a^ ± 0.06	3.53 ^a^ ± 0.04	3.65 ^a^ ± 0.02	3.81 ^a^ ± 0.08	3.94 ^a^ ± 0.03	3.81 ^b^ ± 0.04	3.90 ^a^ ± 0.03	3.87 ^b^ ± 0.04	3.90 ^a^ ± 0.06	3.87 ^b^ ± 0.03	3.74 ^a^ ± 0.18
**L672**	3.17 ^a^ ± 0.08	3.35 ^a^ ± 0.35	3.42 ^a^ ± 0.04	3.11 ^a^ ± 0.40	3.58 ^b^ ± 0.02	3.78 ^a^ ± 0.02	3.82 ^b^ ± 0.02	3.98 ^a^ ± 0.05	3.79 ^a^ ± 0.09	4.04 ^a^ ± 0.09	3.45 ^a^ ± 0.66	3.95 ^a^ ± 0.01	3.63 ^a^ ± 0.37
**L793**	3.53 ^a^ ± 0.06	3.29 ^a^ ± 0.39	3.51 ^a^ ± 0.03	3.32 ^a^ ± 0.33	3.61 ^a,b^ ± 0.02	3.77 ^a^ ± 0.04	3.83 ^b^ ± 0.01	3.83 ^b^ ± 0.05	3.80 ^a^ ± 0.05	3.89 ^b^ ± 0.01	3.72 ^a^ ± 0.16	3.92 ^a^ ± 0.01	3.67 ^a^ ± 0.25
*p*	*0.056*	*0.537*	*0.116*	*0.295*	*0.014*	*0.575*	*0.001*	*0.008*	*0.135*	*0.022*	*0.416*	*0.003*	*0.241*

Different letters represent significant differences batches at the same cold storage time (*p* ≤ 0.05).

## Data Availability

The original contributions presented in the study are included in the article, further inquiries can be directed to the corresponding author.
